# MAINTAINING HEALTHY LIFESTYLE: RACIAL AND GENDER DIFFERENCES IN PHYSICIANS’ ADVICE TO OLDER ADULTS

**DOI:** 10.1093/geroni/igz038.516

**Published:** 2019-11-08

**Authors:** Asif Iqbal, Abolade Oladimeji, Eva Kahana, Boaz Kahana

**Affiliations:** 1 Case Western Reserve University, Cleveland, Ohio, United States; 2 Cleveland State University, Cleveland, Ohio, United States

## Abstract

Older adults are often excluded from physicians’ preventive health promotion recommendations. The influence of patient race and gender on the physician-patient relationship in maintaining good health has been extensively researched, but little is known about the influence of patient race and gender on physicians’ health promotion advice to the elderly. This study explored whether patient race and gender influence primary care physicians’ health promotion advice to older adults. The sample of 536 respondents (µage = 75.51, 75.2% female) was obtained from a NCI funded study of “Health Care Partnerships in Cancer Prevention and Care of Aged. Respondents were randomly selected community dwelling older adults who attend Senior Center programs, sponsored by Area Agencies on Aging (AAA) (Kahana, Lee, Kahana, Langendoerfer, & Marshall, 2015). Multivariate logistic regression analyses were used to control for sociodemographic and health factors while accounting for a complex sampling design. African American patients had greater odds than whites for receiving recommendations to eat a healthy diet (OR = 1.62) and exercise more (OR = 1.83). Elderly women reported fewer recommendations to eat a healthy diet (OR = .81) & exercise more (OR = .45) than did men. Notably, respondents of both races and genders believed that maintaining a healthy lifestyle is very effective. These findings demonstrate that African American and female older adults receive differential health promotional advice from physician and suggest the need for raising physician awareness about the value of prevention advice for all patients.

